# Knowledge and practices of fundoscopy among general practitioners in Qassim Province, Saudi Arabia, for the management of diabetic retinopathy and diabetic macular edema: A cross-sectional study

**DOI:** 10.1177/2050312119900863

**Published:** 2020-01-15

**Authors:** Sultan H Al-Rashidi, Faris S Al-Thunayyan, Khalid A Alsuhaibani, Abdulmajeed A Alharbi, Khalid A Alharbi

**Affiliations:** Department of Ophthalmology, College of Medicine, Qassim University, Buraidah, Saudi Arabia

**Keywords:** DR, DME, fundoscopy, general practitioners, Qassim, Saudi Arabia

## Abstract

**Objectives:**

Blindness is one of the most widespread final pathways of diabetic retinopathy and its associated diabetic macular edema. The general practitioners are the first to encounter these diabetic patients. Fundoscopy is now considered as an ideal way for the diagnosis of patients with diabetic retinopathy. Therefore, this study was undertaken to know the ability and skills of general practitioners for the diagnosis and management of patients with diabetic retinopathy and diabetic macular edema.

**Methods:**

This study was conducted in all major cities in Qassim province of Saudi Arabia during January to May 2017. A validated questionnaire was used to assess the general practitioners’ knowledge and practice for the management of diabetic retinopathy and diabetic macular edema. Questions related to referrals, diagnosis, and treatment options to diabetic retinopathy and diabetic macular edema were asked to the general practitioners.

**Results:**

Of 96 general practitioners, 76 returned the questionnaire with a response rate of 79.2%. Only 26.3% general practitioners referred patients with type 1 diabetes to ophthalmologists as per guidelines set by the American Academy of Ophthalmology, whereas 74% of general practitioners showed good knowledge for referring patients with type 2 diabetes to ophthalmology clinics. Lack of knowledge was also noticed for the treatment of diabetic retinopathy, as only 36.8% of general practitioners replied positive for dilated fundus examination option, whereas 78.9% of general practitioners chose laser photocoagulation as a treatment option. Similar response from them was observed for patients with diabetic macular edema. Furthermore, data also showed years in practice of general practitioners was well correlated with their knowledge for the management of diabetic retinopathy and diabetic macular edema.

**Conclusion:**

The general practitioners included in this study showed lack of knowledge in handling patients with diabetic retinopathy and diabetic macular edema. Therefore, refresher courses are needed that highlight the acquisition of their skills in fundoscopy.

## Introduction

Diabetes mellitus (DM) is considered to be one of the most threatening disorders for human worldwide.^1,2^ In Saudi Arabia, its prevalence is the highest in the Middle East and the third in the world.^2^ DM has also been associated either directly or indirectly with abnormalities of heart, blood vessels, eyes, kidney, or nerves.^1,2^ Not only these, it also has comorbidity with various other disorders.^3–5^ Diabetic retinopathy (DR) is one of the most common causes of irreversible blindness, and its prevalence among patients with DM is 34.6%.^2,3^ The prevalence of DR in Riyadh, Saudi Arabia, was reported to be 31.3%.^2^ Studies in other cities of Saudi Arabia also showed high prevalence, that is, Taif and Alhasa showed nearly 33%,^6,7^ whereas Madinah was reported to be the highest for DR prevalence of 36%.^8^ Hyperglycemia, dyslipidemia, and hypertension are either directly or indirectly associated with DR and may play a role in its onset.^3,9^ Importantly, duration of DM plays a key role in the progression or onset of DR in both type 1 diabetes (T1D) and type 2 diabetes (T2D) patients.^3,9^ According to the International Clinical Diabetic Retinopathy Disease Severity Scale, DR is classified into two stages: non-proliferative and proliferative.^3^ Non-proliferative stage includes microaneurysms, intraretinal hemorrhages, venous dilation, and cotton wool spots, whereas proliferative stage includes one or more of neovascularization and vitreous hemorrhage.^3,9^ Diabetic macular edema (DME) is one of the major complications of DR and is now considered as one of the major leading causes of visual impairment.^10^ Regarding diagnosis of DR and DME, revealing of microaneurysm in the posterior portion of the eye by ophalmoscopy with or without a dilating agent is considered the initial sign of DR.^10–13^ In addition, other methods are time-consuming, expensive, and invasive as fluorescein angiography, but more accurate in detecting the vascular changes in established DR.^10–14^ Moreover, optical coherence tomography (OCT) is non-invasive, quick, and easy and is now considered the gold standard in detecting DR changes even before becoming clinically obvious.^10–14^ Laser photocoagulation is used to heal two complications which are neovascularization of retina and severe macular edema.^10–14^ Only severe cases of non-proliferative DR can be treated with laser photocoagulation because of high possibilities of progression to proliferative DR,^10–14^ whereas intravitreal treatment with anti-vascular endothelial growth factor (VEGF) agents have been replaced by macular laser for DME.^10–14^ Moreover, steroids have been proved to raise the intraocular pressure; therefore, steroids have now been considered as the second-line therapy.^14^ Furthermore, surgical solutions have also been used for some specific DR patients. As example, vitrectomy has been used in DR and DME patients with fractional retinal detachment, fractional macular edema, or vitreous hemorrhage.^10–14^

General practitioners (GPs) are key followers of the diabetic care network,^15,16^ and their awareness levels are important in planning strategies to prevent the onset and the management of DR and DME.^15–17^ DR or DME presents characteristic changes in the fundus of the eye. These changes can be observed before the clinical manifestations of this disorder.^15–18^ Subsequently, the values of fundoscopy have been recognized, and every GP has been expected to be able to use the ophthalmoscope.^15–21^ Therefore, this study was designed to analyze the knowledge and practices of GPs working in Qassim province of Saudi Arabia for the initial screening of patients with DR and its associated DME using fundoscopy.

## Methods

**Figure 1.**
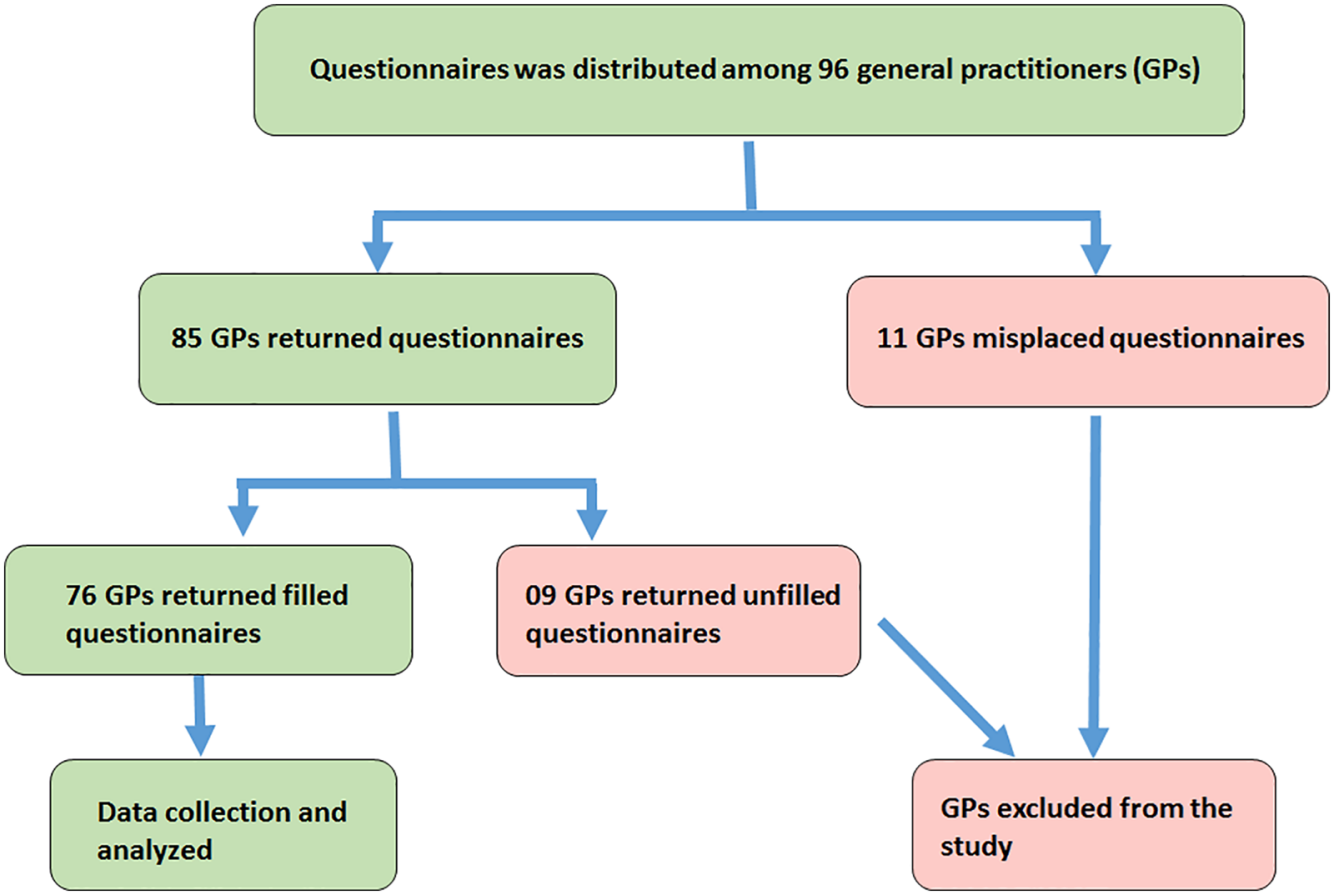
Flow chart of questionnaire distribution and data collection.

This cross-sectional study was conducted in all major cities of Qassim province of Saudi Arabia, including Buraidah, Onaizah, Bukariyah, Ar Rass, and Al Khabra, from January to May 2017. GPs working in all over this region were randomly selected and were interviewed by all authors to fill a questionnaire. A recently published questionnaire was used with modifications.^15^ The required sample size was calculated using the online link https://www.surveysystem.com/sscalc.htm. The complete details of questionnaire distribution among GPs and details of justification of data collection are described in Figure 1. An informed consent was obtained from all GPs included. All GPs were instructed to provide the answers randomly without involving any textbook or colleagues. The complete demographic details of all studied GPs are given in Table 1. The study was approved by the Local Ethics Committee of College of Medicine (approval no. QUCOM#017), Qassim University, Saudi Arabia, and informed consent was taken from all GPs. Statistical analysis was carried out using Graph Pad Prism-5 (San Diego, CA, USA), and p-value less than 0.05 was considered significant. Values are shown as mean ± standard error of mean (SEM) unless otherwise stated.

**Table 1.** Demographic details of studied general practitioners.

Parameters	Specification	n	%
GPs’ response	Total GPs/studied GPs	96/76	79.2
Gender	Male	44	57.9
	Female	32	42.1
Nationality	Saudis	14	18.4
	Egyptians	21	27.6
	Sudanese	23	30.3
	Syrians	3	3.9
	Jordanians	3	3.9
	Pakistanis	6	7.9
	Indians	2	2.6
	Others	4	5.2
Practice (years)	<5	8	10.5
	5–10	19	25.0
	11–20	21	27.6
	>25	28	36.8

GPs: general practitioners.

## Results

**Figure 2.**
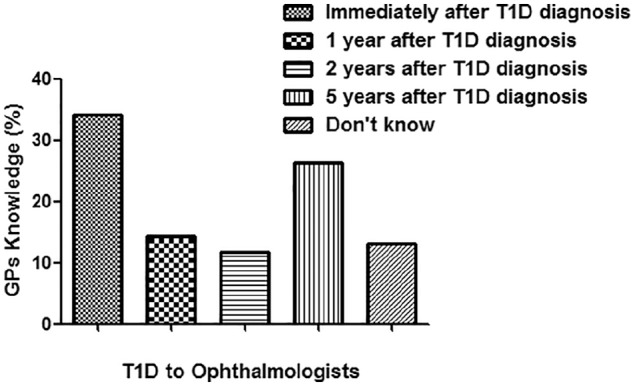
Knowledge of general practitioners (GPs) for the screening of patients with type 1 diabetes (T1D) for referring to ophthalmologists.

**Figure 3.**
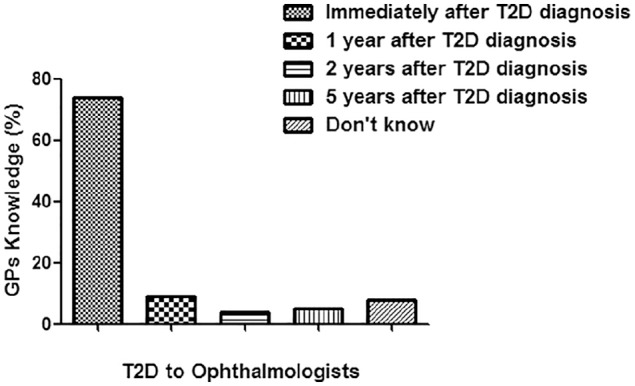
Knowledge of general practitioners (GPs) for the screening of patients with type 2 diabetes (T2D) for referring to ophthalmologists.

**Figure 4.**
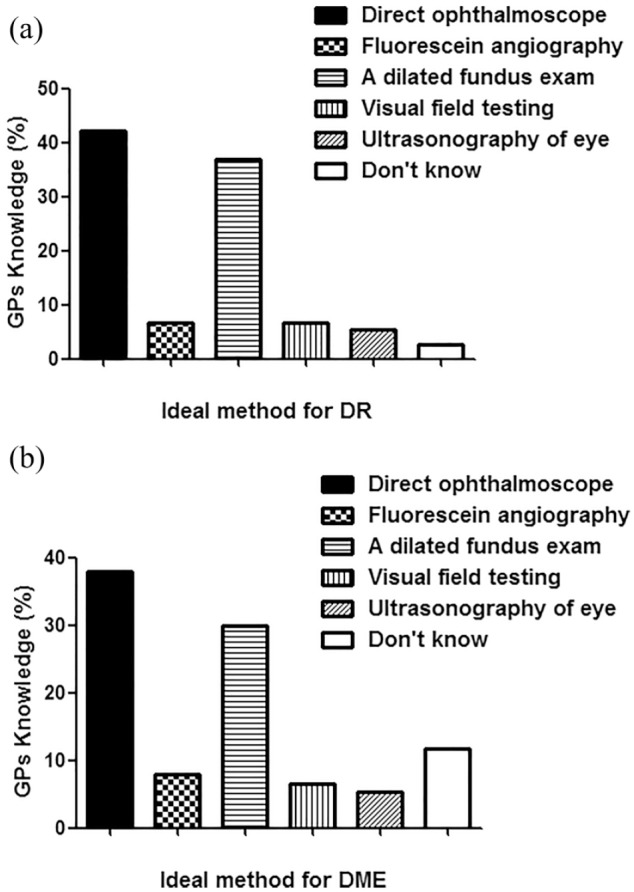
Ideal method of general practitioners (GPs) for the evaluation of patients with (a) diabetic retinopathy (DR) and (b) diabetic macular edema (DME).

**Figure 5.**
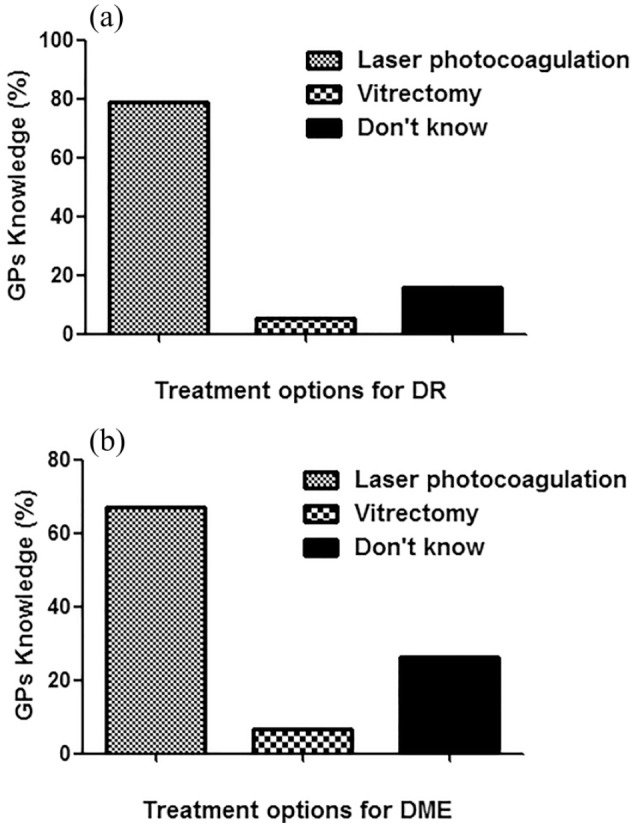
Knowledge of general practitioners (GPs) for the treatment options for patients with (a) diabetic retinopathy (DR) and (b) diabetic macular edema (DME).

Of 96 GPs, 76 returned the filled questionnaire with the response rate of 79.2%. The details of their response are summarized in Figure 1. This cross-sectional study showed that GPs working in Qassim region lack knowledge of screening and follow-up of patients with T1D. Of the 76, only 20 GPs referred patients with T1D to ophthalmologists after 5 years of T1D diagnosis as per diabetic screening guidelines.^22^ Moreover, 23 GPs referred patients with T1D at the time of diagnosis, which showed lack of their knowledge to handle T1D patients. Eleven GPs referred T1D patients after 1 year of diagnosis, and nine GPs referred T1D patients to ophthalmologists after 2 years of diagnosis of T1D. However, 10 GPs responded to the option “didn’t know,” which means they did not know at which stage they should refer the T1D patients to ophthalmologist’s clinics. Knowledge of GPs in percentage for the screening of T1D patients to ophthalmologists is shown in Figure 2. The knowledge of GPs for the screening of T2D patients for referring to ophthalmologists was found to be little bit satisfied as 56 of the 76 GPs referred T2D patients in accordance with the guidelines of T2D.^22^ Seven GPs referred T2D patients after 1 year of diagnosis, three GPs referred patients to ophthalmologists after 2 years of diagnosis of T1D, and four GPs referred patients after 5 years of diagnosis. However, six GPs were unaware at which stage they refer the T2D patients to ophthalmologist’s clinics. Knowledge of GPs in percentage for the screening of T2D patients to ophthalmologists is summarized in Figure 3. We also asked question on the ideal method for the evaluation of DR; only 28 GPs gave a positive reply for choosing dilated fundus examination, which is in accordance with the guidelines set for DR patients.^22^ However, 32 GPs selected direct ophthalmoscope, 5 GPs chose visual field testing, 4 GPs selected fluorescein angiography, another 5 GPs chose ultrasonography of the eye, and the rest did not respond (Figure 4(a)). Furthermore, we also asked the same question but on DME; 23 GPs chose dilated fundus examination, whereas 29 GPs selected direct ophthalmoscope, 5 GPs chose visual field testing, 6 GPs selected fluorescein angiography, 4 GPs chose ultrasonography of the eye, and the rest 9 GPs did not respond (Figure 4(b)). Knowledge of GPs in percentage for selecting the best method for evaluating patients with DR and DME is shown in Figure 4. Lack of GPs’ knowledge was also noticed when we asked a question on the treatment options for DR patients; 60 GPs chose laser photocoagulation as a treatment option for DR patients, 4 GPs selected vitrectomy, and the rest 12 GPs were unaware about LASIK, intravitreal injections, and anti-VEGF or steroid therapies as treatment for retinopathy patients (Figure 5(a)). Almost, similar response was received from GPs when we asked the same question on DME; 50 GPs chose laser photocoagulation as a treatment option for DME patients, 5 GPs selected vitrectomy, and the rest 20 GPs were unaware about LASIK, intravitreal injections, and anti-VEGF or steroid therapies as treatment for DME patients (Figure 5(b)). Furthermore, the knowledge of GPs was also determined in correlation with their practice experience in years, and the data are summarized in Table 2. The data clearly showed that practice experience was positively correlated with their knowledge gain in handling the studied patients.

**Table 2.** Knowledge of general practitioners with their practice experience.

Practice (years)	GPs (%)	Knowledge (%)		
		Poor	Moderate	High
<5	10.5	62.5	21.5	16.0
5–10	25.0	52.6	36.8	10.5
11–20	27.6	38.1	47.6	14.3
>25	36.8	28.6	53.6	17.9

GPs: general practitioners.

## Discussion

This is the first study conducted in all major cities of Qassim province of Saudi Arabia, including Buraidah, Onaizah, Bukariyah, Ar Rass, and Al Khabra, to test the knowledge of GPs for the screening of patients with DR or DME using fundoscopy. Diabetes-associated complications have now become major health problems in all over the world, and the cause of their onset seems to be multifactorial.^1,23–26^ DR is one of its major complications, and its prevalence rate is continuously on the rise in all over the globe including Saudi Arabia.6–8,15 Furthermore, retinopathy in diabetic patients is also associated with the number of other complications, including DME, which is now considered to be the most common problem associated with retinopathy patients.^10^ Now it is well established that the DME frequently occurred in patients with retinopathy at any stage and becomes one of the main reasons to produce the complete blindness.^10^ Therefore, the management of retinopathy as well as DME at the initial stage is extremely important. GPs are key followers of the diabetic care network at the initial phase of screening to prevent the onset of DR and its associated DME.^19–21^ DR and DME both present characteristic changes in the fundus of the eye. These changes are observed before the clinical manifestations of these disorders;^3,6,27^ therefore, every GP is expected to handle these patients by fundoscopy.^1–3^ In Saudi Arabia, the minimum training requirement for the ophthalmology residency is 4 years, and it includes basic science courses followed by the practice of fundoscopy. After completion of this residency program, the health practitioners should be able to identify the patients with DR and also DME. Therefore, in this study, we have tested the knowledge and practices of GPs working all over the Qassim province of Saudi Arabia for the handling of DR and DME patients using fundoscopy. This cross-sectional study is actually in line of the recently conducted study in Riyadh, Saudi Arabia, by Al Rasheed and Adel.^15^ They reported that the knowledge of fundoscopy for DR among physicians working in primary care centers was poor.^15^ Furthermore, Onua and Fiebai^28^ conducted their study in Nigeria, where 125 GPs participated to measure the level of knowledge and practice of fundoscopy. Only 28% of them had good knowledge in which they were capable of answering more than 75% of the questions, while only 15% of them had good practice. Seventy-eight GPs showed good knowledge of referring the patient to ophthalmologist, while 11 of them worked in facilities where no fundoscopy was available.^28^

In this study, the GPs working all over Qassim region were randomly selected and were asked to fill a questionnaire. Of all the selected GPs, the majority of them (79.2%) returned the filled questionnaire. The American Academy of Ophthalmology (AAO) recommended that the first fundus examination in patients with T1D should be performed after 5 years of its diagnosis.^22^ By following the same AAO recommendations, this cross-sectional study noticed that GPs working in Qassim region lack knowledge of screening and follow-up of T1D patients as only 26.3% of GPs referred T1D patients to ophthalmologists after 5 years of T1D diagnosis and the rest failed to respond correctly. These data clearly indicate lack of knowledge of GPs for the handling of T1D patients in terms of referring to the ophthalmology clinics. Furthermore, the knowledge of the same GPs for the screening of T2D patients for referring to ophthalmologists was also investigated and was found to be little bit satisfactory as 73.7% of GPs referred T2D patients in accordance with the guidelines of T2D, which is immediately after the diagnosis of T2D.^22^ Furthermore, we also asked question on the ideal method for the evaluation of DR and DME; only few of them gave a positive reply for choosing dilated fundus examination, which is now considered to be the best method for the diagnosis of DR and DME and also suggested by AAO guidelines.^3,10,22,27^ By following the same AAO recommendations, lack of GPs’ knowledge was also noticed when we asked a question on the treatment options for DR and DME patients as the majority of them were not aware about intravitreal injections and anti-VEGF or steroid therapies. Moreover, the knowledge of GPs was also analyzed in correlation with their practice experience in years, and the data revealed a positive correlation between the years in practice and their knowledge gain in handing the DR or DME patients.

In short, the data clearly reveal that fundoscopy is an underperformed inspection for DR and DME patients among GPs working in Qassim province of Saudi Arabia. Although this study is novel in Qassim and provides important information, there are still few limitations. The ability to generalize our results was limited to the small groups of GPs, and the applied questionnaire was missing the questions on the alternative modern solutions for eye screening of the patients with DM, DR, or DME, such as fundus photography with a non-mydriatic fundus camera and telemedical screening of these patients. In conclusion, this study demonstrated that the knowledge and the practice of fundoscopy for the management of DR and DME among GPs working in Qassim region are poor and far from ideal. Therefore, refresher courses emphasizing the acquisition of the skill in fundoscopy and the provision of ophthalmoscopes for handling patients with DR and forwarding to ophthalmology clinics are needed.

## References

[1] Forbes JM Cooper ME Mechanisms of diabetic complications. Physiol Rev 2013; 93(1): 137–188. 23303908 10.1152/physrev.00045.2011

[2] Elhadd TA Al-Amoudi AA Alzahrani AS Epidemiology, clinical and complications profile of diabetes in Saudi Arabia: a review. Ann Saudi Med 2007; 27(4): 241–250. 17684435 10.5144/0256-4947.2007.241 PMC6074292

[3] Rosenberg JB Tsui I Screening for diabetic retinopathy. N Engl J Med 2017; 376(16): 1587–1588. 28423293 10.1056/NEJMe1701820

[4] Ansari NA Rasheed Z Non-enzymatic glycation of proteins: from diabetes to cancer. Biomed Khim 2010; 56(2): 168–178. 21341505 10.18097/pbmc20105602168

[5] Farhan J Al-Shobaili HA Zafar U, et al Interleukin-6: a possible inflammatory link between vitiligo and type 1 diabetes. Br J Biomed Sci 2014; 71(4): 151–157. 25562992 10.1080/09674845.2014.11669980

[6] Al Ghamdi AH Rabiu M Hajar S, et al Rapid assessment of avoidable blindness and diabetic retinopathy in Taif, Saudi Arabia. Br J Ophthalmol 2012; 96(9): 1168–1172. 22790436 10.1136/bjophthalmol-2012-301874

[7] Khan AR Wiseberg JA Lateef ZA, et al Prevalence and determinants of diabetic retinopathy in Al Hasa region of Saudi Arabia: primary health care centre based cross-sectional survey, 2007–2009. Middle East Afr J Ophthalmol 2010; 17(3): 257–263. 20844683 10.4103/0974-9233.65502 PMC2934719

[8] El-Bab MF Shawky N Al-Sisi A, et al Retinopathy and risk factors in diabetic patients from Al-Madinah Al-Munawarah in the Kingdom of Saudi Arabia. Clin Ophthalmol 2012; 6: 269–276. 22368446 10.2147/OPTH.S27363 PMC3284208

[9] Yau JW Rogers SL Kawasaki R, et al Global prevalence and major risk factors of diabetic retinopathy. Diabetes Care 2012; 35: 556–564. 22301125 10.2337/dc11-1909 PMC3322721

[10] Gundogan FC Yolcu U Akay F, et al Diabetic macular edema. Pak J Med Sci 2016; 32: 505–510. 27182271 10.12669/pjms.322.8496 PMC4859054

[11] Ferris FL III How effective are treatments for diabetic retinopathy. JAMA 1993; 269(10): 1290–1291. 8437309

[12] Nentwich MM Ulbig MW Diabetic retinopathy—ocular complications of diabetes mellitus. World J Diabetes 2015; 6(3): 489–499. 25897358 10.4239/wjd.v6.i3.489 PMC4398904

[13] Lee SJ McCarty CA Sicari C, et al Recruitment methods for community-based screening for diabetic retinopathy. Ophthalmic Epidemiol 2000; 7(3): 209–218. 11035555

[14] Mukamel DB Bresnick GH Wang Q, et al Barriers to compliance with screening guidelines for diabetic retinopathy. Ophthalmic Epidemiol 1999; 6(1): 61–72. 10384685 10.1076/opep.6.1.61.1563

[15] Al Rasheed R Al Adel F Diabetic retinopathy: knowledge, awareness and practices of physicians in primary-care centers in Riyadh, Saudi Arabia. Saudi J Ophthalmol 2017; 31(1): 2–6. 28337055 10.1016/j.sjopt.2017.01.001 PMC5352944

[16] Jacques CH Jones RL Houts P, et al Continuing medical education on diabetes by primary care physicians. Diabetes Educ 1991; 17(4): 269–273. 2049980 10.1177/014572179101700408

[17] Khandekar R Shah S Al Lawatti J Retinal examination of diabetic patients: knowledge, attitudes and practices of physicians in Oman. East Mediterr Health J 2008; 14(4): 850–857. 19166168

[18] Preti RC Saraiva F Junior JA, et al How much information do medical practitioners and endocrinologists have about diabetic retinopathy. Clinics (Sao Paulo) 2007; 62(3): 273–278. 17589667 10.1590/s1807-59322007000300011

[19] Wiggins Michael N Landes Reid D Bhaleeya Swetangi D, et al Primary care physicians’ knowledge of the ophthalmic effects of diabetes. Can J Ophthalmol 2013; 48(11): 265–268. 23931464 10.1016/j.jcjo.2013.03.011 PMC3863606

[20] Muecke JS Newland HS Ryan P, et al Awareness of diabetic eye disease among general practitioners and diabetic patients in Yangon, Myanmar. Clin Exp Ophthalmol 2008; 36(3): 265–273. 18412597 10.1111/j.1442-9071.2008.01724.x

[21] Lazaridis EN Qiu C Kraft SK, et al Same eyes, different doctors: differences in primary care physician referrals for diabetic retinopathy screening. Diabetes Care 1997; 20(7): 1073–1077. 9203439 10.2337/diacare.20.7.1073

[22] American Academy of Ophthalmology Retina/Vitreous Panel. Preferred Practice Pattern® guidelines. Diabetic retinopathy. San Francisco, CA: American Academy of Ophthalmology; 2017, www.aao.org/ppp

[23] Farhan J Alghasham A Zafar U, et al Impact of anti-glutamic acid decarboxylase-65, anti-insulin and anti-tyrosine phosphatase autoantibodies on disease activity in type 1 diabetes patients. J Diab Res Clin Met 2013; 2: 24.

[24] Rasheed Z Al-Shobaili HA Alzolibani AA, et al Immunological functions of oxidized human immunoglobulin G in type 1diabetes mellitus: its potential role in diabetic smokers as a biomarker of elevated oxidative stress. Dis Markers 2011; 31(1): 47–54. 21846949 10.3233/DMA-2011-0803 PMC3826706

[25] Tripathi T Rasheed Z The oxidative by-product, hydroxyl radical, damaged immunoglobulin-G in patients with non-insulin dependent diabetes mellitus. Bratisl Lek Listy 2010; 111(9): 477–484. 21180260

[26] Rasheed Z Ali R Reactive oxygen species damaged human serum albumin in patients with type 1 diabetes mellitus: biochemical and immunological studies. Life Sci 2006; 79(24): 2320–2328. 16945391 10.1016/j.lfs.2006.07.041

[27] Viswanath K McGavin DD Diabetic retinopathy: clinical findings and management. Community Eye Health 2003; 16(46): 21–24. 17491851 PMC1705856

[28] Onua AA Fiebai B Knowledge and practice of fundoscopy among medical doctors in Port Harcourt, Nigeria. Open J Ophthalmology 2016; 6: 164–169.

